# Epileptic Seizures or not, that is the question: a case report

**DOI:** 10.1192/j.eurpsy.2022.1001

**Published:** 2022-09-01

**Authors:** B. Wildenberg, D. Pereira, I. Carvalho, N. Madeira

**Affiliations:** 1 Centro Hospitalar e Universitário de Coimbra, Coimbra, Portugal, Department Of Psychiatry, Coimbra, Portugal; 2 Faculty of Medicine, University of Coimbra, Institute Of Psychological Medicine, Coimbra, Portugal; 3 Centro Hospitalar e Universitário de Coimbra (CHUC), Department Of Neurology, Coimbra, Portugal

**Keywords:** Nonepileptic seizure, Epileptic seizure

## Abstract

**Introduction:**

Psychogenic nonepileptic seizures (PNES) consist of paroxysmal changes in responsiveness, movements, or behaviour that superficially resemble epileptic seizures.

**Objectives:**

Presentation of a clinical case of a PNES in a patient with a diagnose of secondary epilepsy, illustrating the relevance of an adequate evaluation, differential diagnosis, and intervention.

**Methods:**

Description of the clinical case, with brief literature review and discussion. A search was conducted on PubMed and other databases, using the MeSH terms “nonepileptic seizure”, and “epileptic seizure”.

**Results:**

We report the case of a 45-year-old female patient, brought to the emergency department because of tonic axial and limb nonsynchronous movements, closed eyes, long duration, with immediate awareness, no desaturation, tongue bite, facial flushing, dyspnoea or sphincter incontinency. She was medicated with clonazepam 1 mg and levetiracetam 1000 mg ev. TC-CE had no acute alteration. Bloodwork had no other major alteration except valproic acid below therapeutic levels (her usual medication, along with other antiepileptic drugs, antidepressant and antipsychotic). The antecedents of the patient: mild intellectual disability and an accidental traumatic brain injury in infancy, with secondary epilepsy. She was transferred to Psychiatry department. No electroencephalogram (EEG) was realized, because she had a recent one confirming PNES, and many other emergency observations with the diagnosis of PNES.

**Conclusions:**

This clinical case showcases the diagnostic difficulties that clinicians face when there is an overlap in symptoms, emphasizing the need to combine patient history, witness reports, clinician observations, and ictal and interictal EEG to help distinguish these different clinical identities.

**Disclosure:**

No significant relationships.

